# Ferulic Acid-NLC with *Lavandula* Essential Oil: A Possible Strategy for Wound-Healing?

**DOI:** 10.3390/nano10050898

**Published:** 2020-05-08

**Authors:** Claudia Carbone, Carla Caddeo, Maria Aurora Grimaudo, Daniela Erminia Manno, Antonio Serra, Teresa Musumeci

**Affiliations:** 1 Laboratory of Drug Delivery Technology, Department of Drug Sciences, University of Catania, viale A. Doria 6, 95125 Catania, Italy; teresa.musumeci@unict.it; 2 Department of Scienze della Vita e dell’Ambiente, University of Cagliari, via Ospedale 72, 09124 Cagliari, Italy; caddeoc@unica.it; 3 Departamento de Farmacología, Farmacia y Tecnología Farmacéutica, I+D Farma (GI-1645), Facultad de Farmacia and Health Research Institute of Santiago de Compostela (IDIS), Universidade de Santiago de Compostela, 15782 Santiago de Compostela, Spain; maria.aurora.grimaudo@sergas.es; 4 Dipartimento di Matematica e Fisica, University of Salento, 73100 Lecce, Italy; daniela.manno@unisalento.it (D.E.M.); antonio.serra@unisalento.it (A.S.)

**Keywords:** *Lavandula x intermedia “Sumian”*, complementary and alternative medicine, combined delivery, cryo-TEM, cytocompatibility, 2,2-diphenyl-1-picrylhydrazyl (DPPH), migration test

## Abstract

Nowadays, an increasing interest in combinatorial drug delivery systems is emerging, highlighting the possibility of exploiting essential oils (EO) for topical applications. This work aimed at developing nanostructured lipid carriers (NLC) for the combined delivery of ferulic acid and *Lavandula* EO, whose beneficial effects in wound-healing processes have been widely reported. Homogeneous (polydispersity index, PDI < 0.2) nanoparticles with a small size (<150 nm) and a high encapsulation efficiency (>85%) were obtained. The co-presence of ferulic acid and *Lavandula* EO, as compared to synthetic isopropyl myristate-based NLC, increased nanoparticles’ stability, due to higher ordering chains, as confirmed by morphological and physicochemical studies. An enhanced cytocompatibility was observed when combining ferulic acid and *Lavandula* EO, as confirmed by in vitro studies on fibroblasts. Furthermore, the combined delivery of ferulic acid and *Lavandula* EO significantly promoted cell migration with higher effectiveness in respect to the free drug solution and the carrier without the EO. Taken all together, our results suggest a potential combined effect of the antioxidant ferulic acid and *Lavandula* EO co-delivered in lipid nanoparticles in promoting cell proliferation and migration, representing a promising strategy in the treatment of wounds.

## 1. Introduction

The possibility of accelerating the wound-healing processes still represents a challenge for researchers all over the world. In the pharmaceutical technology field, combinatorial drug delivery systems that simultaneously transport two or more active compounds to the targeted site in the human body could represent a promising strategy for improving the effectiveness of traditional drugs [1,2,3,4,5,6,7]. Additionally, complementary and alternative medicines (CAMs) are also attracting increasing interest for the treatment of a great variety of human conditions [8]. In this field, the use of essential oils (EOs) as medical indications of aromatherapy has expanded, suggesting the potentiality of EOs in topical applications, due to their many activities (such as antibacterial, antifungal, or antioxidant) and because they may help improve drug efficacy [9,10]. In particular, wound-healing represents one target for topical application of EOs, offering the possibility of a simple approach with reduced side effects in combination with evidence-based drugs. A correlation between the levels of reactive oxygen species (ROS) and the treatment of skin diseases related to epithelial lesions has been observed, suggesting that the excess of ROS transcends their initial beneficial effects, thus highlighting the potential key role of antioxidants in the treatment of chronic wounds, characterized by an inflammatory phase [11,12]. Among antioxidants, ferulic acid has been demonstrated to promote the wound-healing process when loaded into thermosensitive chitosan-based hydrogels [13] and a micelle-nanogel [14]. Ferulic acid (4-hydroxy-3-methoxycinnamic acid, FA) belongs to the phenolic acid group commonly found in plant tissues (whole grains, spinach, parsley, grapes, rhubarb, and cereal seeds such as wheat, oats, rye, and barley) [15]. FA is considered a superior antioxidant with low toxicity, due to its many physiological functions, i.e., anti-inflammatory, antimicrobial, anticancer, anti-arrhythmic, and antithrombotic activities, as well as antidiabetic and immunostimulant properties. It also reduces nerve cell damage and may help to repair damaged cells [16]. Furthermore, Sangeeta and co-workers have reported the ability of FA to promote tissue regeneration in the skin of diabetic rats, which could be related to its ability to inhibit lipid peroxidation and increase catalase, superoxide dismutase, and glutathione [17]. However, owing to low solubility, low stability, and short residence time, it is necessary to encapsulate FA into a proper drug delivery system able to protect it and, thus, enhance its bioavailability [18]. Different approaches have been developed for FA encapsulation, such as the use of nanoemulsions [19], lipid nanoparticles [20,21], or polymeric micro- or nanoparticles [22,23,24]. In particular, Souto et al. have underlined the advantages of using lipid nanoparticles for topical applications, due to their special character and adhesive properties, which would offer important advantages in topical formulations [25].

Among the pharmaceutically interesting EOs, different authors have highlighted the ability of *Lavandula* EO, belonging to the *Lamiaceae* family, to promote wound-healing in a rat model [8,26,27,28,29]. In particular, the induction of the expression of collagen types 1 and 3, the enhanced proliferation of fibroblasts, and the wound contraction observed in in vitro studies can be related to the EO major components (linalool and linyl acetate), which possess potent anti-inflammatory, antibacterial, and antioxidant activities [8,26,27,28,29]. Accordingly, Mori and coworkers have reported an increase in collagen synthesis by fibroblasts and the expression of transforming growth factor-β (TGF-β), a key molecule involved in the regulation of fibroblasts proliferation and production of wound granulation tissue, after topical treatment of wounds with *Lavandula* EO [8].

Based on these considerations, we prepared nanostructured lipid carriers (NLC) for the combined delivery of FA and *Lavandula x intermedia “Sumian”* EO, used as intrinsic oily component of the formulation and compared to NLC produced with the commercial synthetic oil isopropyl myristate. The aim of our work was to investigate the influence of the EO on NLC structure and properties and whether a synergistic effect between *Lavandula* and FA would have an impact in the wound-healing processes. Unloaded and FA-loaded NLC were characterized by physicochemical, technological, and morphological studies, to evaluate the nanoparticles’ feature in terms of size, polydispersity, zeta potential, pH, osmolarity, drug release, and antioxidant properties (DPPH assay). Furthermore, NLC physical stability was investigated by using Turbiscan^®^ technology. Both unloaded and FA-loaded NLC were investigated by Raman Spectroscopy and cryogenic transmission electron microscopy (Cryo-TEM), to gain information about the influence of the EO on the lipid organization and nanoparticles’ structure. Furthermore, in vitro biological studies were carried out to evaluate the cytocompatibility of the NLC in fibroblasts and the wound-healing and wound-contracting properties. 

## 2. Materials and Methods

### 2.1. Materials

Kolliphor RH40 (Polyoxyl 40 hydrogenated castor oil) was kindly provided by BASF Italia S.p.a. (Cesano Modena, Italy), while Labrafil (Oleoyl Macrogol-6 Glycerides) was a gift from Gattefossé Italia s.r.l. (Milano, Italy). Softisan 100 (Hydrogenated Coco-Glycerides) was purchased from IOI Oleo GmbH (Oleochemicals, IOI group, Hamburg, Germany). *Lavandula x intermedia “Sumian”* EO was kindly provided by Exentiae s.r.l. (Catania, Italy). Isopropyl myristate (IPM) was purchased from Farmalabor (Canosa di Puglia, Italy), while trans-Ferulic Acid (FA) and 2,2-diphenyl-1-picrylhydrazyl (DPPH) were provided by Sigma-Aldrich (Milano, Italy).

### 2.2. Nanoparticle Preparation

NLC were prepared by using the previously reported PIT (Phase Inversion Temperature) method [20]. Samples were prepared by using a very low surfactant mixture concentration (<7% *w/V* of the mixture Kolliphor R40/Labrafil), in which the solid lipid Softisan was added and combined in the ratio 1:2.5 with the liquid oil IPM (NLCa) or *Lavandula* EO (NLCb) (Supplementary Materials Table S1). FA-loaded NLC were prepared by melting the drug (0.5% *w/V*) into the lipid phase of NLC, prepared with IPM (FA-NLCa) or *Lavandula* EO (FA-NLCb). In order to purify the colloidal suspensions from the excess of surfactants and the non-encapsulated drug, each formulation was centrifuged at 13,000 rpm for 2 h at 1 °C, using an ultracentrifuge (SL16R Centrifuge, Thermo Scientific, Rodano, Italy) equipped with a fixed body rotator. The pellet was separated from the supernatant and vortexed (Heidolph Reax 2000, VWR, Milan, Italy) for 60 s.

### 2.3. Photon Correlation Spectroscopy (PCS)

All NLC were analyzed by PCS after a 1:20 dilution (50 µL) in 1 mL of ultra-purified water, to determine mean particle size (Zave), polydispersity index (PDI), and zeta potential (ZP), by the use of a Zetasizer Nano S90 (Malvern Instruments, Malvern, UK). Each formulation was prepared 6 times, and each measure is the mean value of at least three measurements ± standard deviation (SD).

### 2.4. Osmolarity and pH 

Osmolarity values of the samples were determined by an osmometer (Osmomat 3000, Gonotec, Berlin, Germany), previously calibrated with ultra-purified water and physiological solution. A pH meter (Mettler Toledo, Milano, Italy) was used to detect the pH values of the samples.

### 2.5. Stability Studies by Turbiscan^®^ AG Station

Stability studies of the formulations were carried out by using an optical analyzer Turbiscan^®^ Ageing Station (TAGS, Formulaction, L’Union, France) and consisted of the analysis of concentrated dispersions based on Static Multiple Light Scattering technology. Turbiscan^®^ AGS has been previously reported as a reliable technology to evaluate instability phenomena related to particle aggregation and/or migration [30,31,32,33,34,35]. TAGS is equipped with an ageing station and consists of a robot with three thermo-regulated blocks for the storage of 54 samples. NLC (20 mL) are loaded into a cylindrical glass cell and stored in the Turbiscan^®^ at 25.0 ± 1.0 °C for 60 days. The entire height of the sample cell (65 mm longitude) is scanned through the detection head, which is composed of a pulsed near-infrared light source (λ = 880 nm), two synchronous transmission (Tr), and back scattering (BS) detectors, acquiring T each 40 µm (1625 acquisitions in each scan). The T detector receives the light that crosses the sample (at 180° from the incident beam). Turbiscan^®^ measures the variation of the particles’ volume fraction (migration) or diameter (coalescence), resulting in a variation of T signals, and this is an important way to evaluate and detect possible processes of destabilization, also giving information on the type of destabilization. The physical stability of unloaded NLCa and NLCb was analyzed in terms of variation of backscattering profiles (ΔBS). Samples stored in the ageing station at 25 °C were analyzed by PCS after 2 months of storage, in order to evaluate the occurrence of particle aggregation phenomena, together with data obtained by Turbiscan^®^.

### 2.6. Morphological Analysis by Cryo-TEM

Morphology and size of the prepared NLC were determined by cryogenic transmission electron microscopy (Cryo-TEM). All observations were performed by using a Hitachi 7700 electron microscope (Tokyo, Japan), at a temperature of 105 K and an acceleration voltage of 100 KV. The samples were vitrified as described in previous studies [32,36]. Briefly, a drop of solution containing NLC was deposited on copper grids covered by an amorphous carbon film. After removing the excess solution with filter paper, the sample was vitrified by immersion in liquid ethane held just above its freezing point. Then, the sample was transferred to a Gatan 626 cryoholder. The digital images were acquired with an AMT-XR-81 camera and processed with the EMIP software. Counting and size distribution of NLC were obtained from cryo-TEM images by combining the data obtained with two different magnifications (5k× and 40k×). For each sample, 30 fields were analyzed at a 5k× magnification and 30 fields at a 40k× magnification. In this way, the morphology and the granulometry of particles found in randomly chosen areas have allowed a better individual characterization of the coarse NLC (average diameter greater than 200 nm) and the fine NLC (average diameter lower than 200 nm). More than 500 particles were analyzed in every sample, to make a significant statistical analysis.

### 2.7. Raman Spectrometry

A micro-Raman spectrometer (INVIA, Renishaw, Gloucestershire, UK) was used to perform Raman spectroscopy analysis of the NLC, as previously reported [32,36]. The spectrometer was equipped with a 514.5 nm air-cooled Argon ions laser source and an 1800-lines/mm grating monochromator with RenCam CCD detection, providing a resolution of 1 cm^−1^. The spectral resolution was 2 cm^−1^, and the employed laser power was 15 mW. A 100× long working objective was used to focus the laser beam on the sample to 1 µm spot diameter. The Raman signal from the NLC in fluid was collected in backscattering geometry. The acquisition time of Raman spectra was determined by the intensity of the Raman signals and by the signal-to-noise ratio (about 10 min). The power density of the argon laser was limited to levels that do not affect prolonged irradiation and avoid possible laser heating effects during Raman spectral collection. Data analysis was performed by using Renishaw Wire 2.0 software. Data are reported as the mean of the intensity of 100 accumulation spectra acquired from 5 different regions, with a spatial resolution of 5 microns in each sample.

### 2.8. HPLC-UV Analysis

The high-performance liquid chromatography–UV (HPLC-UV) analysis was performed at room temperature, using a Varian Prostar 230 (Varian, Milan, Italy) equipped with an autosampler Varian 410 and Galaxie software for data elaboration, and a reversed-phase C18 column (Symmetry, 4.6 × 150 mm^2^; Waters, Milan, Italy). A mixture of acetonitrile and 2% acetic acid aqueous solution 19:81 (*v/v*) ratio was used as mobile phase. The samples were pumped at 1 mL/min and monitored at λ 290 nm. Under such conditions, calibration curves in ethanol/water 50:50 *v/v* were previously validated [14]. In order to build a calibration curve, known amounts of FA, in the range 0.5–100 μg/mL were dissolved in ethanol/water 50:50 *v/v*, and the absorption was determined for the standard solutions. The linear regression value was: *R*^2^ = 0.9862. No interference of the other formulation components was observed.

### 2.9. Encapsulation Efficiency (EE%)

The amount of the FA encapsulated in the lipid matrix of NLC was determined after ultracentrifugation, dilution in methanol, vortexing, and filtration (0.22 µm), by using HPLC (see Section 2.8). The encapsulation efficiency (EE%) was calculated from the ratio between the quantity entrapped inside the nanoparticles and the total amount of drug used for their preparation:

EE% = amount of drug entrapped/total amount of drug used × 100.

### 2.10. In Vitro Release Experiments

FA release from FA-NLCa and FA-NLCb was evaluated by using Franz-type diffusion cells (LGA, Berkeley, CA, USA). Before being mounted in Franz-type diffusion cells, 0.75 cm^2^ regenerated cellulose membranes (Spectra/Por CE; Mol. Weight Cut-off 3.5 kDa) were moistened by immersion in water for 1 h at room temperature. The receptor compartment was filled with 4.5 mL of phosphate-buffered saline (PBS, pH 7.4), thermostated at 37 °C, and constantly stirred at 600 rpm. Then, 500 µL of each sample was applied in the donor compartment. The experiments were run for 48 h. At scheduled time intervals (0, 1, 2, 3, 4, 5, 6, 7, 8, 9, 24, and 48 h) 200 µL of the receptor medium was withdrawn and replaced with an equal volume of PBS equilibrated to 37 °C, to ensure pseudosink conditions. Each sample was analyzed by the HPLC method described in Section 2.8, to determine the FA content. 

### 2.11. Antioxidant Activity: DPPH Assay

The antioxidant activity of FA-NLCa and FA-NLCb was assessed by evaluating their ability to scavenge 2,2-diphenyl-1-picrylhydrazyl (DPPH), a stable nitrogen-centered free radical. DPPH was dissolved in methanol (40 µg/mL), and the solution was mixed with 20 µL of each sample (having a drug concentration of 20 μg/mL) and stored in the dark, at room temperature, for 30 min. Thereafter, the absorbance was measured at 517 nm against blank (methanol). The extent of discoloration of the violet color of DPPH methanolic solution, quantified as a decrease in absorbance, depends on the intrinsic antioxidant/radical scavenging activity and concentration of a sample. Antioxidant compounds can neutralize the DPPH radical by either direct reduction via electron donation or by radical quenching via hydrogen atom donation. The DPPH radical scavenging activity of each sample was expressed both as percent antioxidant activity (AA), calculated according to the following formula, where *A* is the absorbance:

$$\mathrm{AA}=\left(\frac{A_{\mathrm{DPPH}}-A_{\mathrm{sample}}}{A_{\mathrm{DPPH}}}\right)\times 100$$

and as Trolox equivalent antioxidant capacity (TEAC). The TEAC values were calculated based on a calibration curve plotted using Trolox (reference standard) at different concentrations (0.1–2 mg/mL). Results were expressed as mg Trolox equivalents/mL solution. TEAC reflects the ability of antioxidant samples to scavenge DPPH radical as compared to Trolox: The higher the TEAC values, the higher the radical scavenging activity of a sample.

### 2.12. Cytocompatibility Test 

Cytocompatibility was evaluated in murine fibroblasts (CCL-3T3, ATCC, Manassas, VA, USA). Fibroblasts were seeded in a 96-well plate (2 × 10^4^ cells/well) cultured in 0.2 mL of DMEM medium (Dulbecco’s modified Eagle’s Medium, 10% *v/v* fetal bovine serum, 1% *v/v* penicillin-streptomycin) and were grown for 24 h at 37 °C/5% CO_2_. Formulations (NLCa, NLCb, FA-NLCa, and FA-NLCb) were diluted with DMEM medium, to test different NLC concentrations in the range 0.4–0.0025% *w/V*, as a function of the solid lipid. Two hundred microliters of each formulation was placed in contact with cells for 24 h at 37 °C/5% CO_2_. Controls included cells cultured in DMEM medium. Fibroblast viability was determined by using the Cell Proliferation Reagent WST-1 (Sigma Aldrich, St. Louis, MO, USA). Briefly, 10 μL of reagent and 0.1 mL of DMEM without serum were added to each well; after 90 min, absorbance was measured at 450 and 650 nm (UV Bio-Rad Model 680 microplate reader, Genzano di Roma, Italy). Fibroblast viability was expressed as the percentage of living cells versus controls (n = 3).

### 2.13. Migration Test 

The wound-closure properties of unloaded (NLCa and NLCb) and FA-loaded NLC carriers (FA-NLCa and FA-NLCb) were investigated, using murine fibroblasts (CCL-3T3, ATCC, USA). Fibroblasts were cultured in DMEM medium (Dulbecco’s modified Eagle’s Medium, 10% *v/v* fetal bovine serum, 1% *v/v* penicillin-streptomycin) and grown for 24 h at 37 °C/5% CO_2_ in μ-dishes (1.5 × 10^4^ cells/chamber, Ibidi, Giardini, Italy) with a cell-free gap of 500 μm. All NLC carriers were diluted with DMEM medium to achieve a FA concentration of 20 μg/mL (dilution factor equal to 200). After 24 h culture, the inner insert between the two chambers was removed, and a 1 mL sample was placed in contact with cells for 48 h at 37 °C/5% CO_2_. Controls included cells not exposed to formulations and cells treated with FA solution (20 µg/mL) in DMEM medium. At scheduled times (0, 24, and 48 h), microphotographs were taken with a Nikon Eclipse TS100 Microscope (Tokyo, Japan) equipped with a microscopic digital camera system Olympus DP12 (Tokyo, Japan), to evaluate cell migration within the gap area. Wound-closure properties were evaluated by using ImageJ software (NIH, Bethesda, MD, USA) to calculate the percentage of gap area at each scheduled time versus *t*_0_.

### 2.14. Statistical Analysis

All data are reported as mean values ± SD. Differences, analyzed by Two-sample Hypothesis Testing (*t*-test), using Origin Software (version 8.5.1), were considered statistically significant for *p* < 0.05.

## 3. Results and Discussion

### 3.1. Physicochemical and Technological Characterization

In order to evaluate the potentiality of the co-delivery of *Lavandula* EO and FA in NLC for the treatment of wounds, we designed two NLC formulations, using the commercial synthetic oil isopropyl myristate (IPM) as liquid oil component (NLCa) or *Lavandula* EO (NLCb). The low-energy phase inversion temperature method used allowed the production of NLC with a small number of surfactants, resulting in small-sized nanoparticles below 150 nm (Table 1). Statistically significant reduced values of particle size (*p* < 0.05) were obtained when using *Lavandula* EO as liquid lipid (NLCb), instead of IPM (NLCa). PDI values lower than 0.2 confirmed the great homogeneity in terms of size distribution, related to the presence of a single peak in nanoparticles’ dimensional class (Table 1) without statistically significant differences between samples. All batches showed negative ZP values, without differences (*p* > 0.05) between the two selected oils, and a significant decrease in presence of FA, probably due to the phenolic acid group. It has been widely reported that ZP can be a useful parameter to predict the stability of a colloidal suspension: Values greater than 25 mV (either positive or negative) ensure the nanoparticles’ repulsions, thus improving the physical stability of the sample. As reported in the literature, a coating layer of positively or negatively charged material (chitosan, stearic acid, Eudragit RS 100, didodecyldimethylammonium bromide, cetyltrimethylammonium bromide, and 1,2-dioleoyloxy-3-(trimethylammonium)propane) can be added to increase ZP values of NLC [32,36,37,38,39,40,41]. In the present study, we aimed to simplify the formulation’s composition without the addition of a coating layer of any charged material, which might affect drug release, as previously reported [32,42]. Thus, we verified whether the occurrence of any possible instability phenomena, due to the low ZP values, would be acceptable for the potential topical application in absence of a coating layer. A statistically significant decrease (*p* < 0.05) in particle size values was observed after drug loading in both FA-loaded NLCa and FA-loaded NLCb, whose diameter decreased by ≈35 nm. Drug loading significantly reduced PDI values, as well (Table 1). As we previously demonstrated, the addition of a drug often induces a particle size reduction, probably related to a better organization of the active compound with other NLC components [20]. In order to remove the excess of surfactants, all samples were purified by ultracentrifugation (2 h, 1 °C, 13,000 rpm). PCS analysis confirmed that the centrifugation step did not induce any variation in NLC features, in terms of size, PDI and ZP, whose values did not significantly change (*p* > 0.05), as reported in Supplementary Table S2. A high encapsulation efficiency of the loaded drug was found in both FA-NLCs, whose EE% values were >85% (Table 1).

A reduction in pH values was observed in FA-loaded NLC, going from physiological pH values to more acidic values (Table 1), suggesting that FA is partially localized on the outer surface of the nanoparticles. Analysis through osmometer demonstrated that osmolarity values were close to physiological values in all formulations (Table 1).

**Figure 1:**
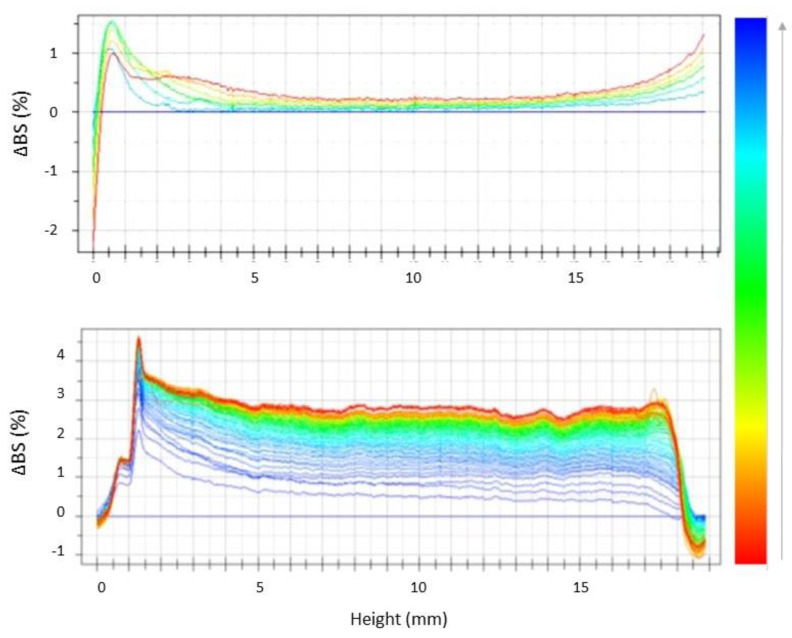
Backscattering profiles (ΔBS) of NLCa and NLCb stored in Turbiscan^®^ for 60 days at 25.0 ± 1.0 °C. Data are presented as a function of time (0–60 days) of sample height (0 to 20 mm) (the direction of analysis time is indicated by the arrow).

In order to evaluate the nanosuspensions shelf life and to compare their physical stability, unloaded NLC were analyzed by using Turbiscan^®^ technology, by storing samples for 60 days at room temperature (25 ± 2 °C). As shown in the graphs of backscattering variations (ΔBS) reported in Figure 1, despite the low ZP values, both colloidal suspensions showed good long-term physical stability with the presence of particle aggregation phenomena, highlighted by variations of BS in the middle of the graph, which were not significant, since ΔBS was <1% for both samples.

It is worth noting that the obtained stability results were in perfect agreement with PCS measurements of NLCb analyzed after two months of storage, showing that, at the 0.05 level of significance, the difference between the population means was not significantly different for mean size and PDI values (Table 2). Size and PDI values significantly increased after two and six months of storage (*p* < 0.05) for NLCa. On the other hand, it is possible to highlight the formation of a peak at the left of the graph (Figure 1), related to the occurrence of a sedimentation phenomenon. It is interesting to observe, thanks to the video files generated by Turbiscan^®^ software, a substantial difference between the two samples, mainly related to the fact that NLCa showed a behavior similar to a deflocculated suspension, which tends to form a more compact sediment at the bottom of the cuvette (Supplementary Video S1). Conversely, NLCb prepared with *Lavandula* EO showed a more stable long-term behavior, similar to a flocculated suspension, showing the tendency of the nanoparticles to agglomerate (Supplementary Video S2) and demonstrating that its stability can be considered acceptable for the intended topical application.

**Figure 2:**
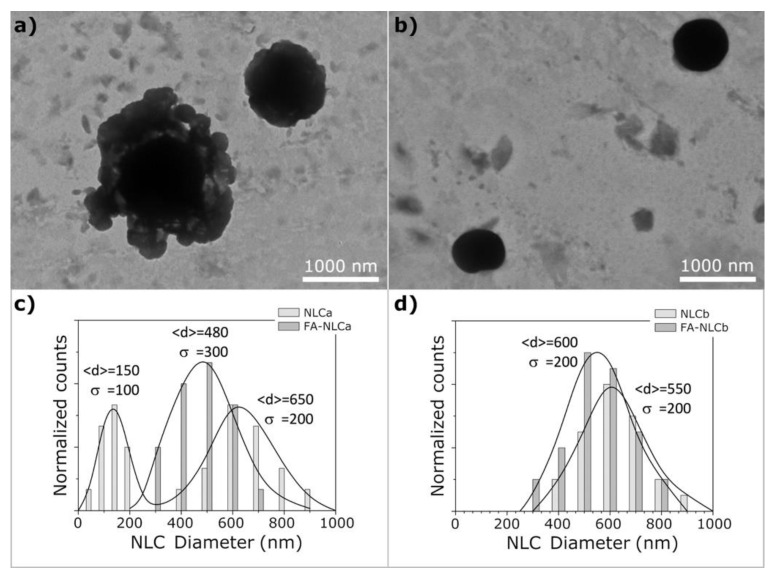
Transmission electron microscopy (TEM) images of NLCa (**a**) and NLCb (**b**). The histograms show the size distribution of the lipid nanoparticles in NLCa (**c**) and NLCb (**d**); continuous curves represent the Gaussian fit of experimental data and the parameters <d> (mean diameter) and *σ* (standard deviation) are also reported.

Cryo-TEM was used to analyze the colloidal suspensions’ morphology very close to their native state, thanks to the vitrification process used for sample preparation. As shown in Figure 2, the presence of the EO in NLCb allowed the production of small spherically shaped particles with a type-II (amorphous) structure (Figure 2b).

**Figure 3:**
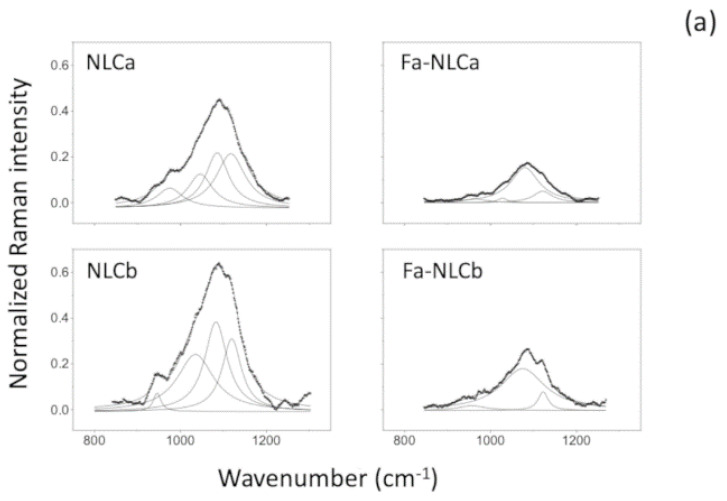

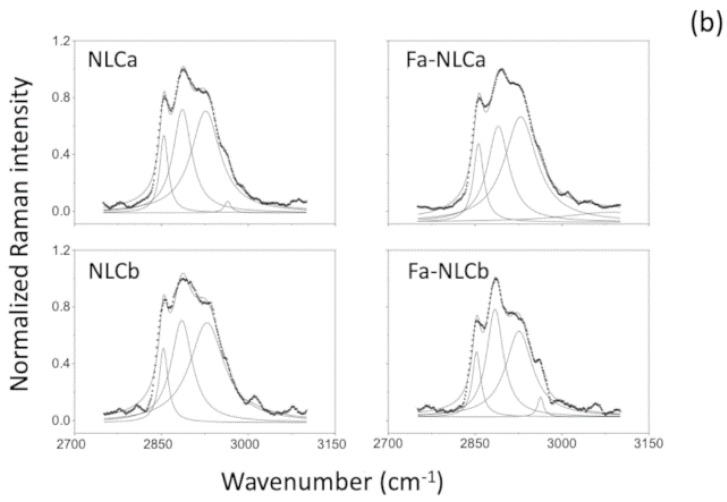
Raman spectra of unloaded and FA-loaded NLCa and NLCb.

NLCa prepared with IPM induced the formation of a similar multiple-type-III NLC, with the presence of grape-like aggregates of very small oil nanocomponents outside the main nanoparticle (Figure 2a). NLCb showed a monomodal particle distribution (Figure 2d), while NLCa showed a bimodal distribution (Figure 2c). These results are in accordance with PCS measurements, suggesting a different organization of raw materials in the NLC systems. Similar results were obtained for FA-loaded NLC, without significant difference compared to the respective unloaded samples (data not shown). Raman spectroscopy was performed to obtain detailed information about the structure of the outer NLC layers. In particular, the structural order evolution of NLC was monitored by analyzing the C–H stretching, C=O stretching, and CH_2_ bending modes. Although no shifts in the frequencies of the Raman transitions arose when comparing the different samples, the relative intensities in significant regions changed remarkably. The survey Raman spectra and the most common vibrational Raman active modes in these NLC formulations are summarized in Supplementary Figure S1 and Supplementary Table S3, respectively. In Figure 3a, a peak of around 1000 cm^−1^, primarily related to C–C stretching motions [43] was reported for all NLC. Both frequency differences and relative intensity changes for these vibrational modes have been used to monitor specific conformational changes in the hydrocarbon chains [44]. Particularly, the 1100 cm^−1^ region showed to be a superposition of the C–C stretching modes for segments of all-trans hydrocarbon conformations. An increase in intensity of the 1115 cm^−1^ band relative to the intensities of the 1065 and/or 1130 cm^−1^ transitions is indicative of a greater fluidity within the hydrocarbon chains; the increase in the 1115 cm^−1^ band originates from the increased intramolecular disorder in the NLC suspensions. In addition, the region around 3000 cm^−1^ of the Raman spectrum consists of a large number of overlapping peaks, containing both fundamental CH-stretch vibrations and Fermi resonance bands [45]. The Raman spectra in the C–H stretching vibration region are shown in Figure 3b. It has been established that the two bands at about 2850 and 2890 cm^−1^ correspond to the symmetric and asymmetric CH_2_ stretching vibrations, whereas the two bands at 2935 and 2965 cm^−1^ correspond to the symmetric and asymmetric CH_3_ stretching vibrations, respectively [46].

The relative intensities of these peaks change notably with changes in hydration state, packing, and conformational order (Table 3). In order to utilize this spectral sensitivity toward the lipid environment, several spectral parameters have been used to empirically describe the order of the lipid bilayer. The ratio *I_1115_/I_1050_* of FA-loaded NLC lowered with respect to unloaded NLC. The lowering was about 17% and 11% for NLCa and NLCb, respectively. The ratio *I_2890_/I_2850_* of FA-loaded NLC increased with respect to unloaded NLC. The increase was not statistically significant (about 2%) for FA-NLCa as compared to NLCa, while a statistically significant increase (about 20%) was observed for FA-NLCb with respect to unloaded NLCb (*p* < 0.05). The peak height ratio *I_2890_/I_2850_* has been used as a marker for chain packing and conformational disorder, where higher values indicate a higher ordering of the chains [45]. Results suggest that FA-NLCb presents the most ordered structure in terms of chain packing, as revealed by the higher values of the peak height ratio I_2890_/I_2850_, related to the presence of a higher ordering of the chains.

**Figure 4:**
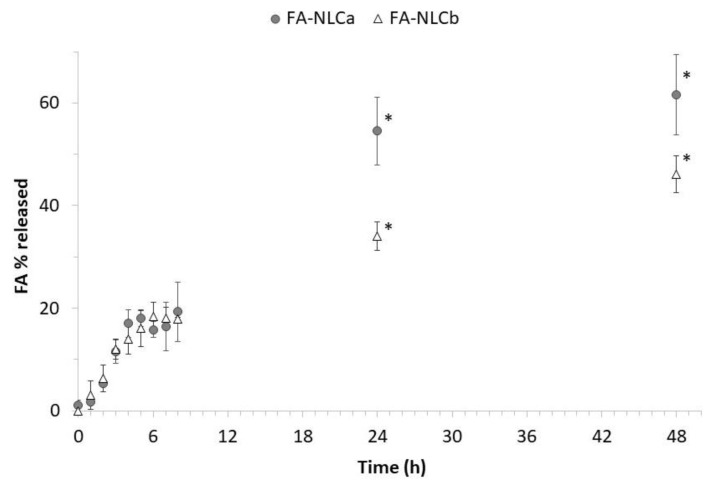
Percentage of ferulic acid (FA) released at different time intervals up to 48 h, from NLC prepared using IPM (FA-NLCa) or *Lavandula* EO (FA-NLCb) as oily phase. Each value is the mean of six independent experiments. * Significance for *p* < 0.05.

In vitro release studies with Franz-type diffusion cells were performed for 48 h on FA-NLCa and FA-NLCb. By comparing the drug-release profiles (Figure 4), it is possible to observe a similar behavior in the first 9 h from the beginning of the experiments, with statistically significant differences between FA-NLCa and FA-NLCb (*p* < 0.05) in the percentage of drug released at 24 and 48 h. It is interesting to highlight that mean drug fluxes were not statistically different, being 0.66 mg/cm/h for FA-NLCa and 0.44 mg/cm/h for FA-NLCb. However, the cumulative amount of drug released at the end of the experiment from FA-NLCb (46% of applied dose) was significantly lower than FA-NLCa (62% of the applied dose). This could be due to the different structure of the nanoparticles, as revealed by cryo-TEM images: The presence of the EO probably allows a better accommodation of the drug molecules in a more ordered structure (type-II), where the drug is solubilized in the oily nano-compartments, thus limiting drug release.

As reported by Blass et al., the use of effective antioxidant molecules can be exploited in the treatment of chronic wounds, to reduce the inflammatory phase [11]. On the other hand, FA’s antioxidant properties have been widely reported [13,15,18,47]. Herein, we aimed to verify if the encapsulation of FA in NLC did not interfere with its intrinsic antioxidant activity. The latter was assessed by the in vitro DPPH assay, which uses the reduction in the concentration of the stable nitrogen-centered free radical DPPH, as a measure of the free radical scavenging potential of the tested samples. The radical is reduced in presence of an antioxidant molecule, which captures the odd electron of DPPH, with a consequent discoloration of the solution, whose degree is related to the scavenging efficiency of the sample, resulting in a decrease or loss of absorbance. As expected, the absence of antioxidant activity was observed for unloaded NLC (data not reported). Interestingly, the DPPH radical was almost completely inhibited by both FA-NLCa and FA-NLCb (90% and 89%, respectively), whose difference was not statistically significant (*p* > 0.05), thus indicating a high antioxidant activity corresponding to ~1 mg/mL of Trolox equivalents (Table 4). 

These findings point out the crucial role of drug incorporation, which was able to protect the drug and preserve the antioxidant activity. These results are corroborated by previous findings, which reported the improvement of the antioxidant activity of drug molecules when incorporated into lipid drug delivery systems [48,49,50].

### 3.2. In Vitro Biological Characterization: Cytocompatibility and Wound-Healing Activities

**Figure 5:**
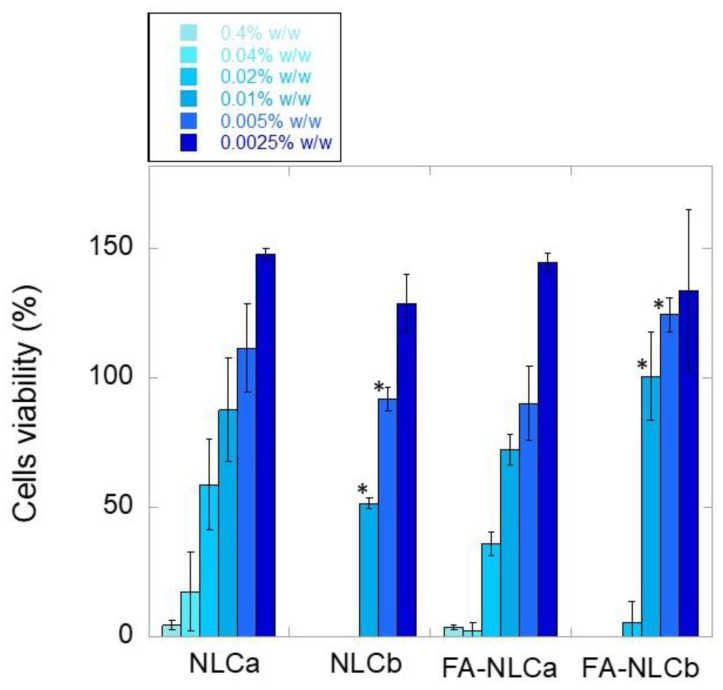
Murine fibroblasts viability after contact for 24 h with unloaded and FA-loaded NLC at different concentrations. Results are reported as the mean value ± SD of three separate experiments, each performed in triplicate. * Significance for *p* < 0.05, comparison between FA-loaded NLC and the respective unloaded NLC.

All the prepared NLC were studied in terms of cell biocompatibility, to evaluate their potential topical application in wound-healing. Fibroblasts were selected as an indicator of cell viability, due to their ability to produce growth factors that control cell proliferation and differentiation, important aspects in wound-healing processes. Both unloaded and FA-loaded NLC were tested at different dilutions, corresponding to solid lipid concentration range of 0.0025–0.4% *w/V* (Figure 5). As reported, NLC showed a good cytocompatibility at a solid lipid concentration equal to or lower than 0.005% *w/V*. Interestingly, FA encapsulation together with the EO (FA-NLCb) improved the cytocompatibility of the NLC, which showed a statistically significant improvement of cell tolerability (*p* < 0.05) at a solid lipid concentration equal to 0.01% and 0.005% *w/V* (Figure 5). The obtained results suggest a potential combined protective effect of the antioxidant drug (25 µg/mL) and *Lavandula* EO (0.0025% *w/V*), whose co-presence in the NLC formulation significantly improved cell viability. Previous findings reported by Picone et al. have showed that the loading of FA (28 mM) into solid lipid nanoparticles prepared with Compritol 888ATO provided better results in terms of cell viability, due to the reduction of ROS production [18]. The FA protective effect observed for FA-NLCb, but not for FA-NLCa, could be ascribed to the fact that, even if a very low antioxidant activity was reported in literature for the EO of the Lamiaceae family [51], it is enough to promote a synergistic effect when combined with an antioxidant drug.

Furthermore, it is worth noting that the lowest NLC concentration (0.0025% *w/V*) was shown to stimulate fibroblast proliferation, which is considered an important factor for dermis regeneration [52]. Similar studies on the proliferative effects of EO-loaded NLC have been reported regarding Rosemary EO nanoemulsion on fibroblasts [53]. Furthermore, Alexander et al. have highlighted the potential to exploit the increase in cell proliferation observed loading thymoquinone into NLC, as a strategy to increase the number of cells to cover the scratched wound areas that could be beneficial for the treatment of wounds [54]. Herein, it is interesting to highlight that the proliferative effect occurs when combining FA with the EO. However, it is important to specify that at 24 h, drug release from FA-NLCb was very low, being less than 50% of drug loaded (Figure 4). It is possible that the more-ordered type-II structure of FA-NLCb, responsible for the slower drug release in comparison with FA-NLCa, could positively affect cytocompatibility, maintaining a slow but constant amount of FA available, to exert its protective effect on cells. Nevertheless, considering the small differences observed in terms of Zave and ZP for all the NLC (Table 1), further studies need to be performed to investigate the role that each NLC component could play in the observed fibroblast proliferation.

**Figure 6:**
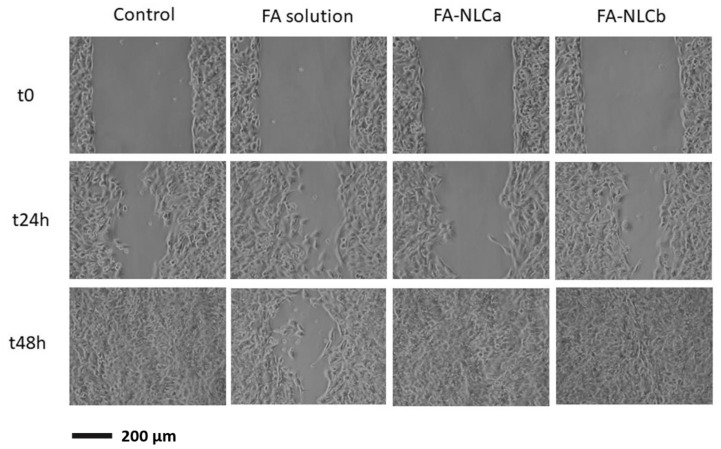
Photographs of the gaps among fibroblast cells at 0, 24, and 48 h after incubation with free FA, FA-loaded NLCa, and FA-loaded NLCb at 37 °C in 5% CO_2_ and 95% relative humidity.

Then, we investigated the wound-closure-promoting properties of FA-loaded NLC in vitro. This wound-healing assay represents a simple and low-cost in vitro method to study directional cell migration. This method simulates cell migration that could occur in vivo during wound-healing processes. Specifically, a “wound” was created in a cell monolayer, and images were captured at the beginning of the experiment and at different time intervals (24 and 48 h). In order to compare the effect of the loaded drug with the free FA, we also tested FA solution prepared in DMEM at the same concentration. As expected, unloaded NLC were not able to stimulate cell migration at different time intervals (Supplementary Figure S2). By comparing FA-NLCa and FA-NLCb performances (fibroblast gap after 24 h incubation reduced to approximately 41% and 63% of the initial fibroblasts gap at *t*_0_ for FA-NLCb and FA-NLCa, respectively), FA-NLCb resulted in being the most efficient formulation in promoting fibroblast migration and wound closure (Figure 6). According to previous findings [55,56], the fibroblast gap after 24 h incubation reduced to ≈46% in the control and a spontaneous wound closure occurred at 48 h. Therefore, compared to the control, Fa-NLCb enhanced cell proliferation and migration at 24 h and completely induced the wound closure after 48 h treatment, with no residual trace of scar that would seem to remain in control and FA-NLCa. Since FA release after 24 and 48 h was lower for NLC prepared with *Lavandula* EO than for FA-NLCa, it is possible that the wound closure occurred thanks to a combined effect of the antioxidant FA and the EO. Therefore, the type-II structure of FA-NLCb created due to the presence of the EO not only seems to affect the nanoparticles’ features and cumulative amount of drug released, as previously discussed, but it could also interfere with the behavior in cells, improving the cytocompatibility and enhancing fibroblast migration. 

Indeed, it has been recently proposed that *Lavandula* EO could promote a more rapid wound closure, probably due to its ability to affect granulation tissue induced by platelet-derived growth factors (PDGFs) and re-epithelialization induced by epidermal growth factors (EGFs) [57]. Our results are also in accordance with findings reported by Ben Djemaa et al., who observed a reduction in the wound area treated with an ointment containing *Lavandula* EO; the authors infer that this effect could be attributed to the cicatrisation, antimicrobial, and anti-inflammatory activities of the EO, related to the presence of monoterpenes [51]. Nevertheless, after 48 h, both FA-loaded NLC resulted in being able to promote cell migration; therefore, it could be suggested that the amount of drug released from FA-NLCa was able to determine the same effects produced by a reduced amount of FA released from FA-NLCb, whose efficacy was probably improved by the presence of the EO. It is interesting to underline that the free drug at the same tested concentration was not able to promote wound closure but behaved worse than untreated cells. This result could be attributed to the ability of FA-NLCa and FA-NLCb to control FA release (Figure 4), while the release of FA in solution is expected to be massive and could negatively interfere with cell metabolism. In addition, drug encapsulation in NLC would also result in the protection of the drug from instability phenomena, which strongly limit its effectiveness [58]. 

Therefore, FA-NLCb resulted the most effective formulation in promoting fibroblast migration and wound closure. Its efficacy could be attributed to the co-presence of FA and *Lavandula* EO, which shows a synergistic effect in promoting cell migration, along with the advantage of using a lower amount of the antioxidant.

## 4. Conclusions

Taken together, our results suggest that FA-loaded NLC prepared using *Lavandula* EO as oily liquid component represents a promising formulation for future application in wound-healing products. Therefore, the developed NLC formulation plays a key role in the sustainable development of topical products in which CAMs can be exploited to improve the antioxidant effectiveness of FA, exploiting its ability to promote cell proliferation and migration in wound-healing.

## Figures and Tables

**Table 1:** Mean particle size (Zave, nm), polydispersity index (PDI), zeta potential (ZP), percentage of encapsulation efficiency (EE%), pH, and osmolarity (mOsm) of unloaded and FA-loaded NLC prepared using IPM (NLCa) or *Lavandula* EO (NLCb). Each value is the average of six different replicates ± standard deviation (SD). * Comparison between NLCa with IPM vs. NLCb with *Lavandula*, EO. ** Comparison of the loaded sample with the respective unloaded NLC, significance for *p* < 0.05.

Batch	Zave (nm) ± SD	PDI ± SD	ZP ± SD	EE%	pH	Osmolarity (mOsm)
NLCa	122.51 ± 5.98 *	0.101 ± 0.007	−4.85 ± 0.15	-	7.21 ± 0.02	0.282 ± 0.005
NLCb	99.88 ± 1.33 *	0.089 ± 0.015	−5.02 ± 0.02	-	7.13 ± 0.05	0.289 ± 0.008
FA-NLCa	87.77 ± 6.30 **	0.167 ± 0.061	−2.53 ± 0.03 *	86.55 ± 0.95	6.02 ± 0.03	0.285 ± 0.008
FA-NLCb	62.86 ± 0.75 **	0.056 ± 0.012	−2.09 ± 0.05 *	87.02 ± 1.98	5.98 ± 0.01	0.301 ± 0.007

**Table 2:** Mean particle size (Zave, nm) and polydispersity index (PDI) ± standard deviation (SD) of unloaded NLCa and NLCb stored in Turbiscan^®^ at 25 °C and analyzed after preparation and after two or six months. Each value is the average of six different replicates ± standard deviation (SD). * Significance for *p* < 0.05, comparison between NLC analyzed at different time intervals.

Sample	Zave ± SD	PDI ± SD
**After Preparation**		
NLCa	122.51 ± 5.98	0.101 ± 0.007
NLCb	99.88 ± 1.33	0.089 ± 0.015
**After 2 Months of Storage**		
NLCa	163.2 ± 0.8 *	0.189 ± 0.006 *
NLCb	107.5 ± 0.5	0.109 ± 0.005
**After 6 Months of Storage**		
NLCa	186.9 ± 0.7 *	0.218 ± 0.009 *
NLCb	157.2 ± 0.9 *	0.101 ± 0.007

**Table 3:** Raman intensity ratios related to C-C stretching vibrational bands, *I_1115_/I_1050_*, and Intensity Raman ratios, *I_2890_/I_2850_*, for the C–H stretching vibrational bands of unloaded and FA-loaded NLC. Values are the mean of the intensity of 100 accumulation spectra acquired from five different regions with a spatial resolution of 5 microns in each sample. SD is <0.02 for all averages. * Significance for *p* < 0.05.

Sample	*I_1115_/I_1050_*	*I_2890_/I_2850_*
NLCa	0.92	1.25
FA-NLCa	0.76	1.27
NLCb	0.89	1.18 *
FA-NLCb	0.79	1.43 *

**Table 4:** In vitro antioxidant activity (AA) of FA-loaded NLC prepared using IPM (FA-NLCa) or *Lavandula* EO (FA-NLCb) as oily phase. DPPH results are expressed as AA (%) and as TEAC (mg/mL) concentration. Results are reported as the mean value ± SD of three separate experiments, each performed in triplicate.

Batch	AA (%)	TEAC (mg of Trolox Equivalents/mL)
**FA-NLCa**	90.8 ± 0.6	1.17 ± 0.005
**FA-NLCb**	89.3 ± 0.9	1.15 ± 0.008

## Supplementary Materials

The following are available online at https://www.mdpi.com/2079-4991/10/5/898/s1. Figure S1: Survey Raman spectra of different analyzed systems. The regions of interest (850–1250 cm^–1^ and 2725–3125 cm^–1^) are enlarged and discussed in the text. Figure S2: Photographs of the gaps among fibroblast cells at 0, 24, and 48 h after incubation (at 37 °C in 5% CO_2_ and 95% relative humidity) with control, unloaded NLCa, and unloaded NLCb. Table S1: Quali-quantitative composition of blank and Fa-loaded NLC prepared using IPM (NLCa) or Lavandula EO (NLCb) as liquid oily phase. Table S2: Mean particle size (Zave, nm), polydispersity index (PDI) and zeta potential (ZP) ± standard deviation (SD) of unloaded and FA loaded NLC measured before and after purification. Table S3: Common vibrational Raman active modes in NLC formulations. Video S1: Turbiscan^®^ analysis of NLCa. The video shows the nanosuspension backscattering profile evolution over seven days of storage at 25 ± 2.0 °C, highlighting the presence of an evident sediment in the deflocculated suspension. File created by Turbisoft-AGS Version 1. Video S2: Turbiscan^®^ analysis of NLCb. The video shows the nanosuspension backscattering profile evolution over seven days of storage at 25 ± 2.0 °C, highlighting the presence of a flocculated suspension with greater stability. File created by Turbisoft-AGS Version 1.
