# Supplementary material for: Ferulic Acid-NLC with Lavandula Essential Oil: A Possible Strategy for Wound-Healing?
Source: Nanomaterials (Basel). 2020 May 8;10(5):898. doi: 10.3390/nano10050898 (PMC7279150; doi:10.3390/nano10050898)
Supplement: Supplementary file 1 [file nanomaterials-10-00898-s001.zip › nanomaterials-743688-SI/nanomaterials-743688-SI.pdf]

nanomaterials

Supplementary Materials

Ferulic Acid-NLC with *Lavandula* Essential Oil: A Possible Strategy for Wound-Healing?

Claudia Carbone [1,*], Carla Caddeo [2], Maria Aurora Grimaudo [3], Daniela Manno [4], Antonio Serra [4], Teresa Musumeci [1]

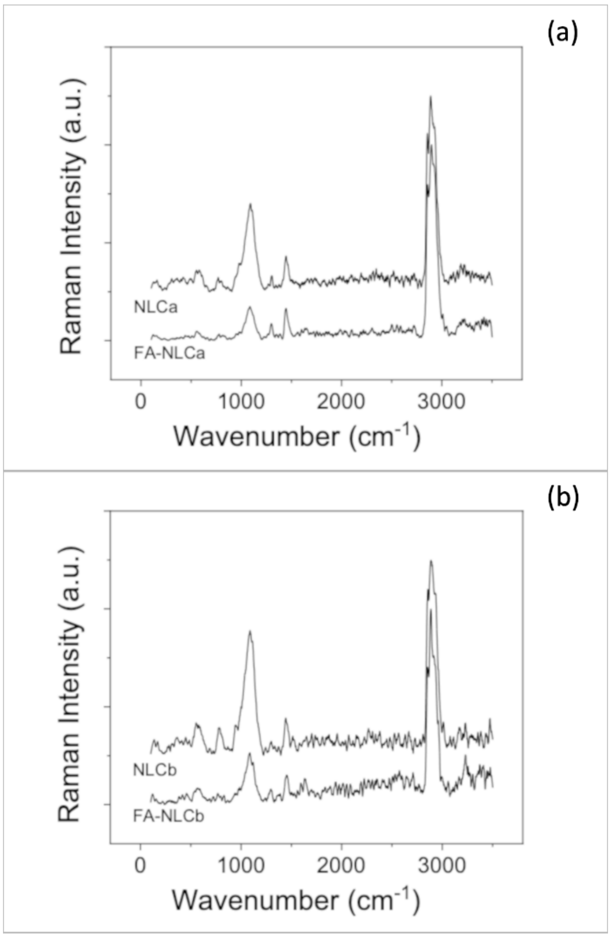

Figure S1. Survey Raman spectra of different analyzed systems. The region of interest (850–1250 cm^{-1} and from 2725–3125 cm^{-1}) are enlarged and discussed in the text.

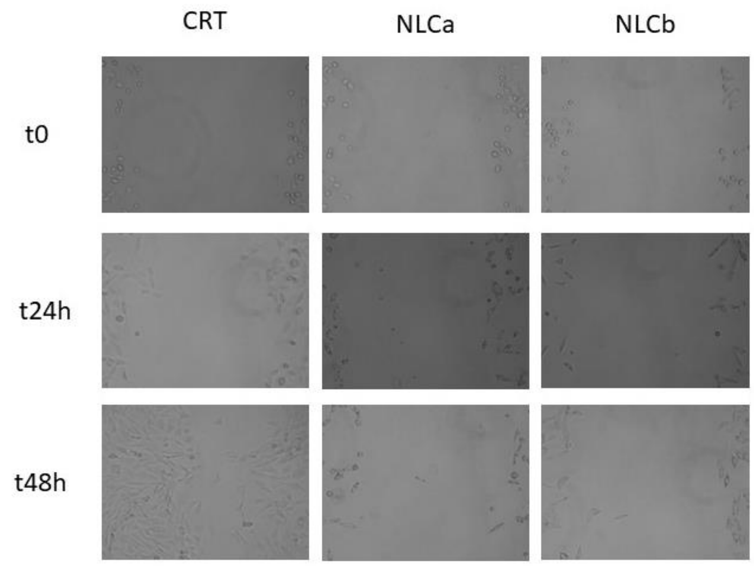

Figure S2. Photographs of the gaps among fibroblast cells at 0, 24 and 48 h after incubation (at 37 °C in 5% CO_2 and 95% relative humidity) with control, unloaded NLCa and unloaded NLCb.

Table S1. Quali-quantitative composition of blank and Fa-loaded NLC prepared using IPM (NLCa) or *Lavandula* EO (NLCb) as liquid oily phase.

**Batch**	**NLCa**	**NLCb**
**Compound**	**% w/V**	**% w/V**
**Kolliphor**	4.5	4.5
**Labrafil**	2.0	2.0
**Softisan**	2.0	2.0
**Oil**	5.0 (IPM)	5.0 (*Lavandula* EO)

Table S2. Mean particle size (Zave, nm), polydispersity index (PDI) and zeta potential (ZP) ± standard deviation (SD) of unloaded and FA loaded NLC measured before and after purification.

Batch	After preparation			After centrifugation		
	**Zave (nm) ± SD**	**PDI ± SD**	**ZP ± SD**	**Zave (nm) ± SD**	**PDI ± SD**	**ZP ± SD**
NLCa	122.51 ± 5.98	0.101 ± 0.007	- 4.85 ± 0.15	125.01 ± 6.2	0.170 ± 0.018	- 5.18 ± 0.15
NLCb	99.88 ± 1.33	0.089 ± 0.015	- 5.02 ± 0.02	98.36 ± 1.40	0.123 ± 0.020	- 5.25 ± 0.02
FA-NLCa	87.77 ± 6.30	0.167 ± 0.061	- 2.53 ± 0.03	95.02 ± 1.65	0.172 ± 0.033	- 3.01 ± 0.02
FA-NLCb	62.86 ± 0.75	0.056 ± 0.012	- 2.09 ± 0.05	71.74 ± 1.85	0.099 ± 0.027	- 2.89 ± 0.03

Each value is the average of six replicates. *Significance for $p < 0.05$, comparison between NLC analyzed before and after centrifugation.

Table S3. Common vibrational modes.

Functional group mode	Approximate wave number (cm^{-1})
$-CH_3$, symmetric and antisymmetric stretch	2920-2960
$-CH_3$, symmetric and antisymmetric stretch	2850-2890
$-PO_2$, symmetric and antisymmetric stretch	1080-1200
-C-C-	1050-1150
-C-O-	1410
-C=O	1720
-COH	870-890
-OH	1080-1090
$-CH_2-$, deformation	1460-1470
